# Comprehensive analysis of miRNA–mRNA regulatory network and potential drugs in chronic chagasic cardiomyopathy across human and mouse

**DOI:** 10.1186/s12920-021-01134-3

**Published:** 2021-11-29

**Authors:** Jiahe Wu, Jianlei Cao, Yongzhen Fan, Chenze Li, Xiaorong Hu

**Affiliations:** 1 Department of Cardiology, Zhongnan Hospital of Wuhan University, No. 169 Donghu Road, Wuchang District, Wuhan, 430071 China; 2 Institute of Myocardial Injury and Repair, Wuhan University, Wuhan, China

**Keywords:** Chronic chagasic cardiomyopathy, Hub gene, miRNA–mRNA regulatory network, Gene function enrichment analysis, PPI network, Bioinformatics analysis

## Abstract

**Background:**

Chronic chagasic cardiomyopathy (CCC) is the leading cause of heart failure in Latin America and often causes severe inflammation and fibrosis in the heart. Studies on myocardial function and its molecular mechanisms in patients with Chronic chagasic cardiomyopathy are very limited. In order to understand the development and progression of Chronic chagasic cardiomyopathy and find targets for its diagnosis and treatment, the field needs to better understand the exact molecular mechanisms involved in these processes.

**Methods:**

The mRNA microarray datasets GSE84796 (human) and GSE24088 (mouse) were obtained from the Gene Expression Omnibus (GEO) database. Homologous genes between the two species were identified using the online database mining tool Biomart, followed by differential expression analysis, gene enrichment analysis and protein–protein interaction (PPI) network construction. Cytohubba plug-in of Cytoscape software was used to identify Hub gene, and miRNet was used to construct the corresponding miRNA–mRNA regulatory network. miRNA-related databases: miRDB, Targetscan and miRWalk were used to further evaluate miRNAs in the miRNA–mRNA network. Furthermore, Comparative Toxicogenomics Database (CTD) and L1000 Platform were used to identify hub gene-related drugs.

**Results:**

A total of 86 homologous genes were significantly differentially expressed in the two datasets, including 73 genes with high expression and 13 genes with low expression. These differentially expressed genes were mainly enriched in the terms of innate immune response, signal transduction, protein binding, Natural killer cell mediated cytotoxicity, Tuberculosis, Chemokine signaling pathway, Chagas disease and PI3K−Akt signaling pathway. The top 10 hub genes LAPTM5, LCP1, HCLS1, CORO1A, CD48, TYROBP, RAC2, ARHGDIB, FERMT3 and NCF4 were identified from the PPI network. A total of 122 miRNAs were identified to target these hub genes and 30 of them regulated two or more hub genes at the same time. miRDB, Targetscan and miRWalk were further analyzed and screened out hsa-miR-34c-5p, hsa-miR-34a-5p and hsa-miR-16-5p as miRNAs regulating these hub genes. Finally, Progesterone, Flutamide, Nimesulide, Methotrexate and Temozolomide were identified to target these hub genes and might be targeted therapies for Chronic chagasic cardiomyopathy.

**Conclusions:**

In this study, the potential genes associated with Chronic chagasic cardiomyopathy are identified and a miRNA–mRNA regulatory network is constructed. This study explores the molecular mechanisms of Chronic chagasic cardiomyopathy and provides important clues for finding new therapeutic targets.

**Supplementary Information:**

The online version contains supplementary material available at 10.1186/s12920-021-01134-3.

## Introduction

Chronic Chagasic Cardiomyopathy (CCC), first reported by Carlos Chagas in 1909, is a myocardial disease caused by a protozoan parasite, *Trypanosoma cruzi* [1, 2]. CCC is characterized by severe cardiac inflammation and myocardial fibrosis and is the leading cause of myocardial diseases and heart failure in Latin America [3]. The incidence of CCC has declined as living conditions have improved, but the current trend of people moving from rural to urban areas complicates the situation [4]. Studies have shown that chagas disease still affects 6–8 million people and kills about 12,000 people a year [5] and the positive rate of *T. cruzi* in serum of Latin American immigrants with non-ischemic cardiomyopathy in the United States ranges from 13 to 19% [6].

CCC often causes symptoms of palpitations or syncope from arrhythmia, chest pain from microvascular dysfunction and heart failure from left ventricular dysfunction.

The pathogenesis of chronic chagas cardiomyopathy is very complex. At present, there are four hypotheses: direct injury caused by parasites [7], inflammatory reaction [8], microvascular injury [9] and autonomic nerve dysfunction [10]. Patients eventually died of malignant arrhythmia (55–65%) in early adulthood, mainly because persistent ventricular tachycardia (VT) degenerated into ventricular fibrillation [11]. There are many studies on CCC, but there is still no effective treatment at present, and the prognosis of chagas cardiomyopathy is very poor.

Animal models of CCC, especially mouse models, have attracted researchers' attention due to the high homologous and identity of human and mouse genome [12, 13]. The research on the similarity of the molecular characteristics of human and mouse diseases has led to the rapid development of disease prognosis and treatment. Studies have shown that RARP1 plays an important role in CCC, which leads to the increase of mtROS and oxidative stress in chagasic myocardium through the decrease of Pol γ-dependent mtDNA content, mtDNA coding gene expression and mitochondrial respiratory function [14]. This indicates that RARP1 may be an important therapeutic target of CCC. Furthermore, TGF-β inhibitor therapy can decreases fibrosis and stimulates cardiac improvement in CCC [15]. The dysregulation of microRNAs (miRNAs), including: miR-19a-3p, miR-21-5p, miR29b-3p, miR-21, miR-146 are reported to be related with CCC development [16–18]. However, the identity of miRNA–mRNA regulations in CCC had not been reported till now.

**Fig. 1:**
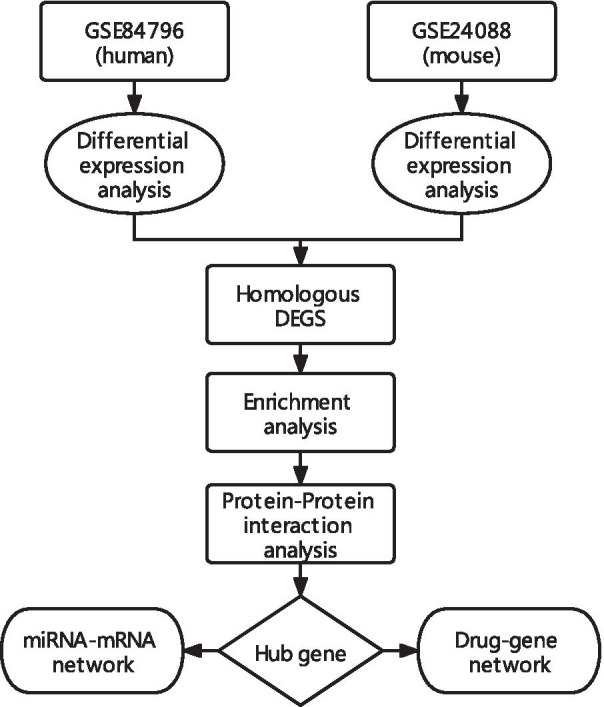
Flowchart of the steps performed in this study

This study was performed to identify the co-differently expressed miRNA–mRNA regulatory networks and developmental programs in CCC between human and mouse. The GSE84796 and GSE24088 datasets were downloaded and homologous genes between human and mouse CCC models were identified and analyzed using bioinformatics analyses. Our study would provide new insights of molecular mechanisms involved CCC, aiming to understand the development and progression of CCC and find targets for its diagnosis and treatment. The workflow of the specific analysis is shown in Fig. 1.

## Methods

### Data resource

The mRNA microarray datasets GSE84796 (human) and GSE24088 (mouse) were obtained from the Gene Expression Omnibus (GEO) database. (https://www.ncbi.nlm.nih.gov/). GSE84796 (human) and GSE24088 (mouse) is based on the GPL14550 platform (Agilent-028004; SurePrint G3 Human GE 8 × 60 K Microarray) and the GPL8938 platform (Duke Mouse 30 k; Oligonucleotide Array Operon V3.0.1). GSE84796 is comprised of 10 CCC samples (patients have severe Chagas Chronic Cardiomyopathy) and 7 control samples (healthy organ donors, these hearts were not used for transplantation due to size mismatch with available recipients). GSE24088 is comprised of 4 CCC samples (C57Bl/6 mice chronically infected with *T. cruzi* (6 months)) and 4 control samples (normal control).

### Identification of DEGs in chronic chagasic cardiomyopathy

The gene expression dataset was downloaded from the abovementioned platform and GEO2R was adopted to identify the DEGs between CCC samples and control samples. The adjusted P-values (adj. P) and log-fold change (FC) in expression were determined. Benjamini–hochberg method with default values was used to correct adj. *P* for potential false positive results. Adj. *P* < 0.05 and |logFC|> 1.0 was set as the specific cut-off criteria of DEGs.

### Annotations of homologous genes across species

The homologous genes between human and mouse were identified using the online database mining tool Biomart [19, 20]. (v2.3.6; https://bioconductor.org/packages/release/bioc/html/biomaRt.html).

### Identification of homologous DEGs between human and mouse

Venn diagram web tool (http://bioinformatics.psb.ugent.be/webtools/Venn/) was used and the identified homologous genes were intersected with genes that had obvious common differential expression in the two datasets. The heat maps of Homologous DEGs were produced using the package g plots of R software (version: × 64 3.2.1) [21, 22].

### Function enrichment analysis of homologous DEGs

The Gene Ontology (GO; http://www.geneontology.org) knowledgebase is the world’s largest source of information on the functions of genes. GO describes the function of gene products in all organisms and identify transcriptome data or characteristic biological properties of high-throughput genomes. Three categories: biological process (BP), cellular component (CC) and molecular function (MF) are its main classification [23]. Kyoto Encyclopedia of Genes and Genomes (KEGG; http://www.kegg.jp/ or http://www.genome.jp/kegg/) is a database resource for understanding high-level functions and utilities of the biological system, such as the cell, the organism and the ecosystem, from molecular-level information, especially large-scale molecular datasets generated by genome sequencing and other high-throughput experimental technologies [24]. The Database for Annotation, Visualization, and Integrated Discovery (DAVID; version6.8; https://david.ncifcrf.gov) was used for enrichment analysis of Homologous DEGs. *P* value < 0.05 was regarded as statistically significant.

### Construction of the protein–protein interaction network and module analysis

In order to better understand the molecular mechanism of CCC, a protein–protein interaction network (PPI network) of Homologous DEGs was constructed. The String database (version10.0; http://string-db.org) was used to perform critical assessments and integrations of protein interactions, including physical (direct) and functional (indirect) associations [25]. Based on the results of the analysis, PPI network was established by Cytoscape software (version 3.7.1). Taken the scores of Maximal Clique Centrality (MCC) algorithm as the criteria, the Cytohubba plug-in of Cytoscape software was used to screen out the top10 Hub genes with high connectivity in the PPI network. What’s more, the significant modules of the PPI network were screened using the plug-in MCODE (the parameters were set to default). The KOBAS (version 3.0; http://kobas.cbi.pku.edu.cn/) database was used to explore the key pathways of these modules [26].

### Construction of the miRNA-hub gene regulatory network

miRNet (https://www.mirnet.ca) is an easy-to-use, miRNA-centric network visual analytics platform aiming to help elucidate microRNA functions by integrating existing knowledge with users' data [27]. miRNet was used to identify miRNAs targeting Hub gene. The miRNA–mRNA network of CCC was established by Cytoscape software (version 3.7.1). miRNA-related datasets miRDB ( http://mirdb.org), Targetscan (version7.2; http://targetscan.org) and miRWalk (http://mirwalk.umm.uni-heidelberg.de/) were used to further evaluate the regulatory relationships predicted by the miRNet database [28–30].

### Hub gene-drug interaction network analysis

The public Comparative Toxicogenomics Database (CTD; http://ctdbase.org/) is a digital ecosystem that relates toxicological information for chemicals, genes, phenotypes, diseases [31]. The hub gene-drug interaction network was constructed using CTD, aiming to search chemotherapeutic drugs that could reduce or increase the expression levels of the hub genes. Briefly, these hub genes were searched in CTD database, and the hub gene-drug interaction networks were visualized by Cytoscape software (version 3.7.1). Besides, these hub genes were also queried using the Connectivity Map online tool (L1000 platform; https://clue.io/l1000-query) [32]. The molecular structure of the drugs predicted by both databases were found in the Drugbank database (http://go.drugbank.com) [33].

## Results

### Identification of homologous DEGs between human and mouse

**Fig. 2:**
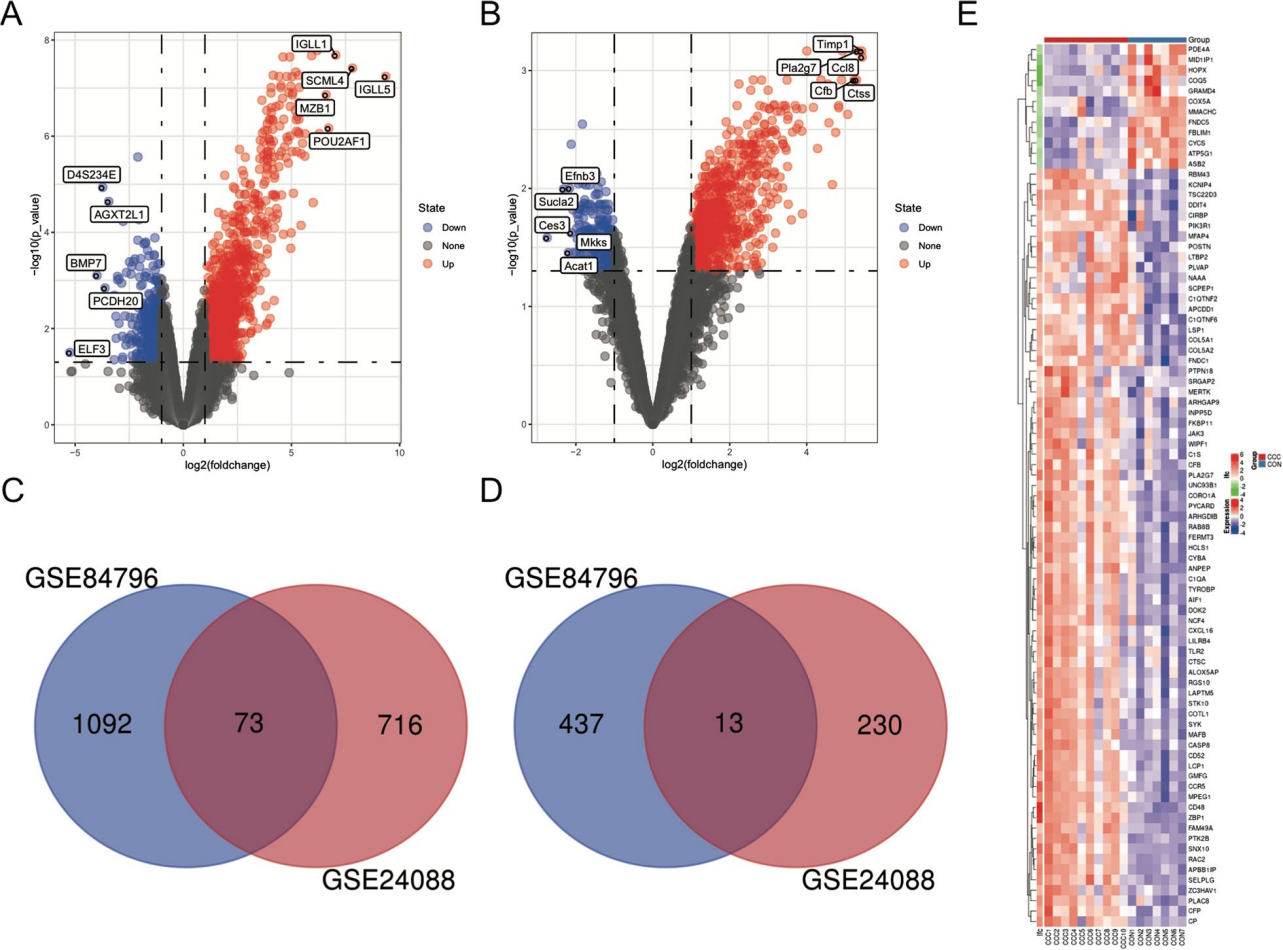
Homologous DEGs in GSE84796 (human) and GSE24088 (mouse). **A** Volcano plots of GSE84796. **B** Volcano plots of GSE24088. For **A** and **B**, differentially expressed molecules were screened under the cut-off criteria |log2FC|> 1 and the adjusted *P* value (*P* < 0.05). **C** Venn diagram of up-regulated Homologous DEGs. **D** Venn diagram of down-regulated homologous DEGs. **E** Heatmap of Homologous DEGs in GSE84796

The mRNA microarray datasets GSE84796 (human) and GSE24088 (mouse) were obtained from the Gene Expression Omnibus (GEO) database. According to the GEO2R software analysis and the screening conditions, DEGs in each GEO dataset were identified. There were 1615 DEGs, including 1165 upregulated and 450 downregulated genes in GSE84796 (human), 1032 DEGs, including 789 upregulated and 243 downregulated genes in GSE24088 (mouse). The volcano plots of the distribution of DEGs in each dataset are shown in Fig. 2A and B. The DEGs are listed in Additional file 1. The top 10 high-expressed DEGs and the top 10 low-expressed DEGs in each dataset are shown in Tables 1 and 2. The online database mining tool Biomart was used to identify the homologous genes. A total of 17,354 homologous genes were identified and these homologous genes between human and mouse are list in Additional file 2. These identified homologous genes were intersected with genes that had obvious common differential expression in the two datasets and 86 Homologous DEGs (Additional file 3), including 73 upregulated and 13 downregulated genes, were identified. Figure 2C and D is the Venn diagram showing this process. Figure 2E is the Heatmap of these Homologous DEGs in GSE84796 (human).

**Table 1:** The top 10 high-expressed DEGs and the top 10 low-expressed DEGs in GSE84796

Datasets	GENE_SYMBOL	Gene ID	logFC	adj.P.Value	Expression
GSE84796 (human)	IGLL5	100423062	9.3527332	5.72E−08	Up
	SCML4	256380	7.8225719	3.85E−08	Up
	IGLL1	3543	7.0467008	2.07E−08	Up
	POU2AF1	5450	6.7202013	6.84E−07	Up
	MZB1	51237	6.6000016	1.38E−07	Up
	CCL5	6352	6.2834279	8.89E−07	Up
	IGJ	3512	6.183125	1.66E−08	Up
	GZMH	2999	6.1234204	6.47E−08	Up
	ZNF683	257101	6.0890868	5.72E−08	Up
	FCRL5	83416	6.0100721	5.72E−08	Up
	ELF3	1999	− 5.2432822	3.15E−02	Down
	RPS4Y1	6192	− 5.1884702	7.90E−02	Down
	RPS4Y2	140032	− 5.1431484	7.72E−02	Down
	NCRNA00185	83869	− 4.605292	4.00E−02	Down
	DDX3Y	8653	− 4.5198597	5.46E−02	Down
	BMP7	655	− 3.9880484	7.84E−04	Down
	D4S234E	27065	− 3.7308209	1.16E−05	Down
	PCDH20	64881	− 3.6182641	1.44E−03	Down
	EIF1AY	9086	− 3.4750827	7.78E−02	Down
	AGXT2L1	64850	− 3.447762	2.28E−05	Down

**Table 2:** The top 10 high-expressed DEGs and the top 10 low-expressed DEGs in GSE24088

Datasets	GENE_SYMBOL	Gene ID	logFC	adj.P.Value	Expression
GSE24088 (mouse)	Ccl8	20307	5.441605	7.70E−04	Up
	Timp1	21857	5.429127	6.84E−04	Up
	Pla2g7	27226	5.320059	6.84E−04	Up
	Ctss	13040	5.296175	1.20E−03	Up
	Cfb	14962	5.233954	1.21E−03	Up
	H2-Aa	14960	5.064829	2.35E−03	Up
	Plac8	231507	4.932353	6.84E−04	Up
	Lilrb4	292594	4.907422	1.20E−03	Up
	Itgb1bp3	69564	4.834374	2.07E−03	Up
	Mpeg1	17476	4.831578	1.99E−03	Up
	Ces3	23491	− 2.746517	2.62E−02	Down
	Sucla2	20916	− 2.321407	1.01E−02	Down
	Acat1	110446	− 2.195231	3.50E−02	Down
	Efnb3	13643	− 2.164247	9.97E−03	Down
	Mkks	59030	− 2.121421	2.38E−02	Down
	Dnaja3	83945	− 2.120821	4.23E−03	Down
	Lrtm1	319476	− 2.076317	1.51E−02	Down
	Gapdh	14433	− 2.068512	3.50E−02	Down
	Asb15	78910	− 2.030904	4.46E−02	Down
	3110057O12Rik	269423	− 1.992578	3.49E−02	Down

### Enrichment analysis for homologous DEGs

**Fig. 3:**
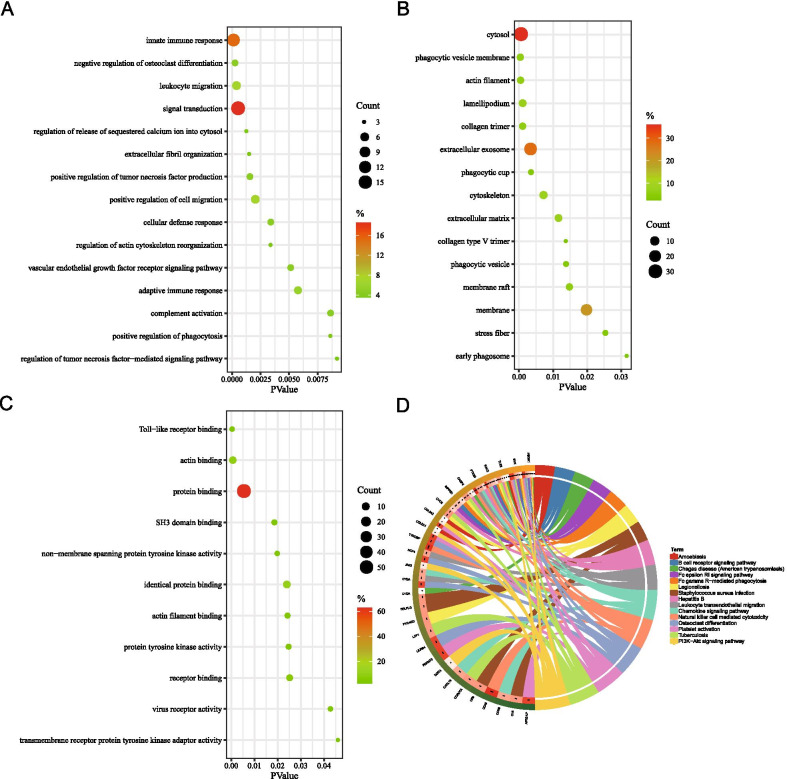
GO and KEGG pathway enrichment analyses. **A** Bubble plot of Homologous DEGs in the term of biological process (BP; TOP15). **B** Bubble plot of Homologous DEGs in the term of cellular component (CC; TOP15). **C** Bubble plot of Homologous DEGs in the term of molecular function (MF; TOP13). **D** Circle plot of KEGG pathway enrichment analysis of Homologous DEGs (TOP15). (*P* < 0.05)

GO knowledgebase and KEGG database were used to characterize the functional roles of the above Homologous DEGs. Figure 3 list the top15 enriched GO terms and KEGG pathways (*p* < 0.05). The BP category of the GO analysis results showed that these Homologous DEGs were significantly enriched in the term of innate immune response, leukocyte migration, signal transduction, positive regulation of cell migration and adaptive immune response (Fig. 3A). For GO CC analysis, the top four significantly enriched terms were cytosol, phagocytic vesicle membrane, actin filament, lamellipodium (Fig. 3B). The top four significantly enriched MF terms included Toll−like receptor binding, actin binding, protein binding, SH3 domain binding (Fig. 3C). Furthermore, Natural killer cell mediated cytotoxicity, Platelet activation, Tuberculosis, Leukocyte transendothelial migration, Chemokine signaling pathway, PI3K−Akt signaling pathway and Chagas disease (American trypanosomiasis) are pathways of significant enrichment in KEGG analysis (Fig. 3D). Detailed analysis results are in Additional file 4.

### Construction of the protein–protein interaction network and identification of hub genes and key modules

**Fig. 4:**
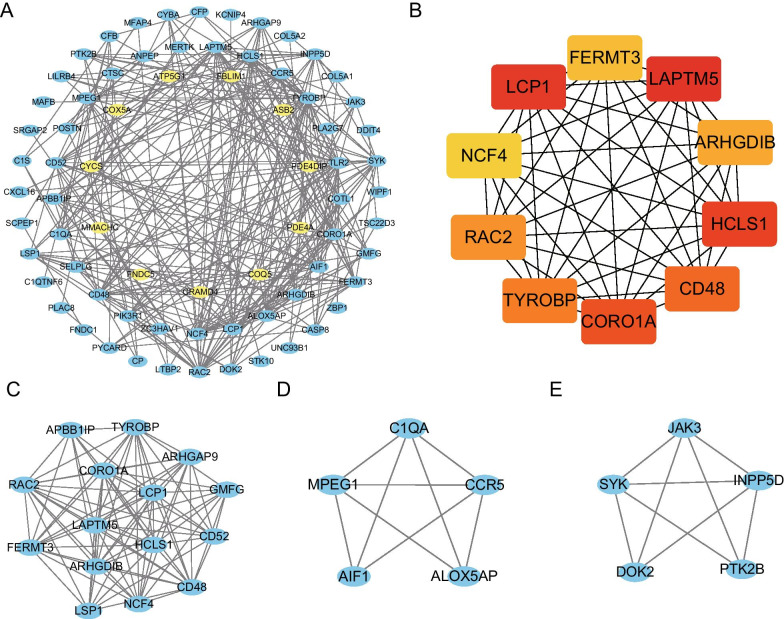
Protein–protein interaction network, hub genes and key modules. **A** The whole PPI Network. The 11 yellow nodes in the network represent down-regulated genes and the 61 blue nodes in the network represent up-regulated genes. **B** PPI network of the 10 hub genes. **C** PPI network of module 1. **D** PPI network of module 2. **E** PPI network of module 3

The Homologous DEGs obtained were introduced into the online database String. After removing the isolated genes without interaction, a PPI network with 72 nodes and 279 edges was established (Fig. 4A). The 11 yellow nodes in the network represent down-regulated genes and the 61 blue nodes in the network represent up-regulated genes. Next, we filtered out the top 10 hub genes using the plug-in Cytohubba in Cytoscape using MCC method. They were LAPTM5, LCP1, HCLS1, CORO1A, CD48, TYROBP, RAC2, ARHGDIB, FERMT3 and NCF4. The PPI network of these 10 hub genes is shown in (Fig. 4B). We identified 3 modules in the whole network on the basis of MCODE (Fig. 4C–E).

**Fig. 5:**
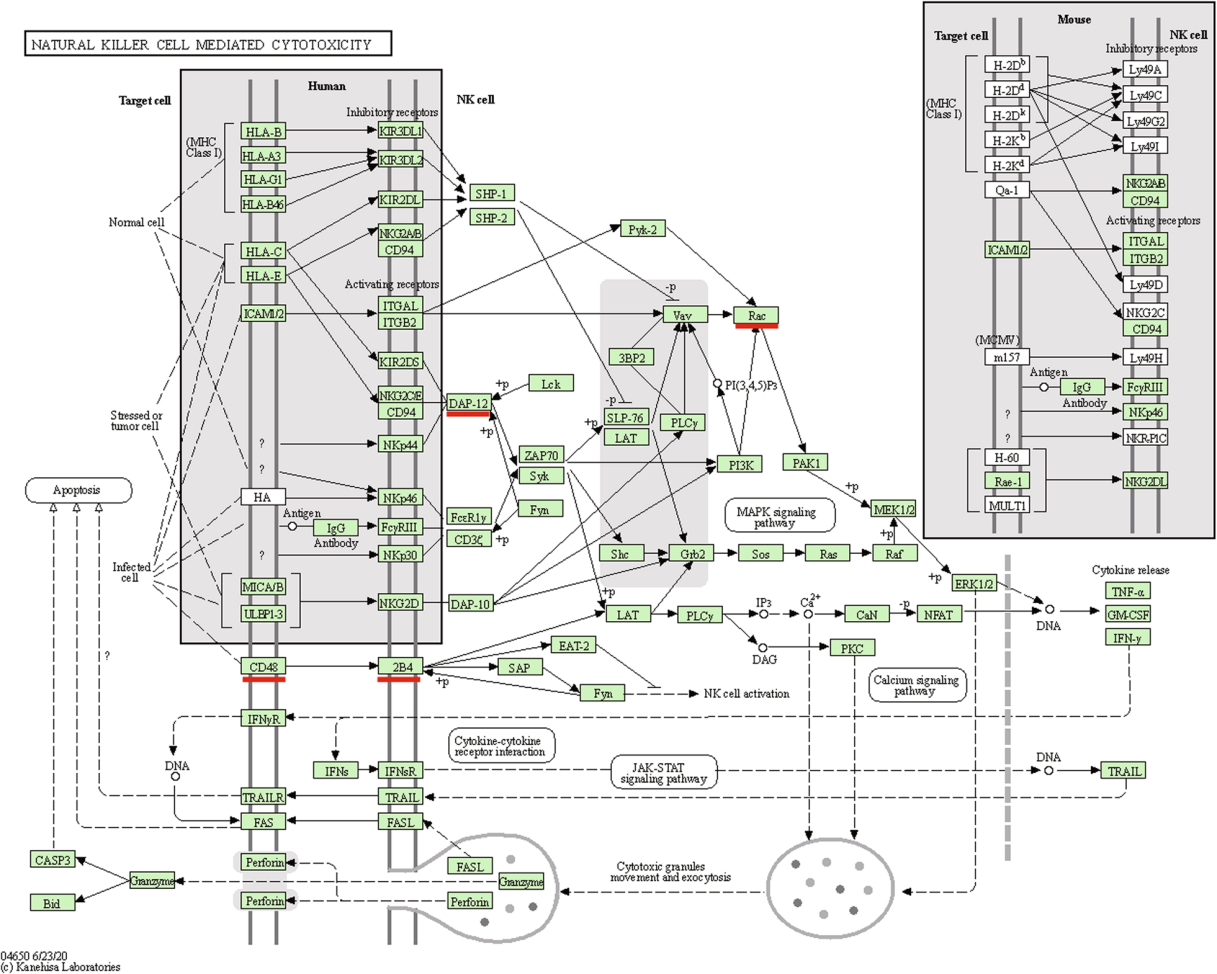
Pathway of Natural killer cell mediated cytotoxicity. The Hub gene (CD48, TYROBP, RAC2) is highlighted by a subscript red segment

Pathway enrichment analysis shows that these 10 hub genes are significantly enriched in the pathway of Natural killer cell mediated cytotoxicity (Fig. 5). Furthermore, PHENSIM simulator (https://phensim.atlas.dmi.unict.it/) was used to analyze these hub genes [34], and it was found that the influence of these hub genes on natural killer cell medicated cytotoxicity signaling pathway was realized by perturbation of MAPK3, SHC2, LAT, SHC1 and other genes. In this pathway diagram, the Hub gene is highlighted by a subscript red segment. In addition, the DEGs of module 1 are significantly enriched in the pathway of Natural killer cell mediated cytotoxicity, Leukocyte transendothelial migration, Platelet activation and Phagosome. The DEGs of module 2 are enriched in Prion diseases, Fc epsilon RI signaling pathway, *Staphylococcus aureus* infection and Pertussis. Furthermore, the DEGs of module 3 are significantly enriched in Fc epsilon RI signaling pathway, B cell receptor signaling pathway, Fc gamma R-mediated phagocytosis and Natural killer cell mediated cytotoxicity. These hub genes and genes of top 3 modules are shown in Table 3.

**Table 3:** The top three significant modules and hub genes in the PPI network

Plug-in	Modules	Nodes	Edges	Genes
MCODE	Module 1	15	89	RAC2, FERMT3, ARHGAP9, LSP1, CORO1A, GMFG, TYROBP, LAPTM, CD52, CD48, NCF4, LCP1, ARHGDIB, HCLS1, APBB1IP
MCODE	Module 2	5	9	C1QA, CCR5, ALOX5AP, MPEG1, AIF1
MCODE	Module 3	5	9	DOK2, INPP5D, JAK3, SYK, PTK2B
CytoHubba	Hub gene	10	45	LAPTM5, LCP1, HCLS1, CORO1A, CD48, TYROBP, RAC2, ARHGDIB, FERMT3, NCF4

### Construction of the miRNA-hub gene regulatory network

**Fig. 6:**
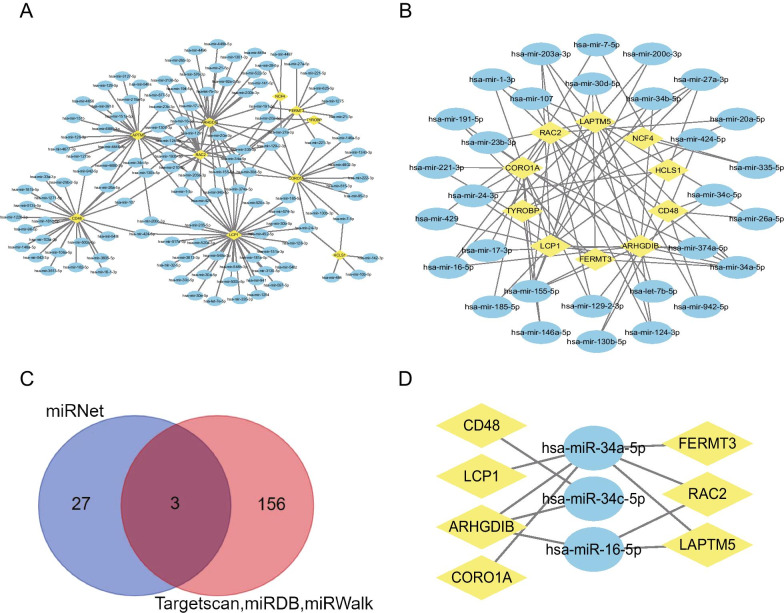
miRNA–mRNA network. **A** The whole miRNA–mRNA Network. **B** miRNAs that simultaneously targeted two or more Hub Genes. **C** Venn diagram of miRNA prediction results. **D** Co-predicted miRNA–mRNA regulatory relationships. The yellow diamond nodes represent Hub Genes; The blue elliptic nodes represent miRNAs

miRNet was used to identify miRNAs targeting Hub genes and a miRNA–mRNA regulatory network with 132 nodes (122miRNAs and 10 hub genes) and 170 edges was established (Fig. 6A). We screened miRNAs that simultaneously targeted two or more Hub genes and constructed another miRNA–mRNA regulatory network with 40 nodes (30miRNAs and 10 hub genes) and 78 edges (Fig. 6B). miRNAs of hsa-miR-34a-5p, hsa-miR-30d-5p, hsa-miR-27a-3p, hsa-miR-24-3p, hsa-miR-203a-3p, hsa-miR-16-5p, hsa-miR-155-5p, hsa-miR-1-3p, hsa-miR-129–2-3p and hsa-miR-124-3p, which targeted 3 or more Hub genes, are screened (Additional file 5). Hsa-miR-34a-5p and hsa-miR-155-5p targets six hub genes simultaneously. We identified these correlations in this miRNA–mRNA network using three miRNA-related online databases, Targetscan, miRDB and miRWalk, and identified 3miRNAs and 11 regulatory relationships (Fig. 6C, D): hsa-miR-34a-5p-LCP1, hsa-miR-34a-5p-ARHGDIB, hsa-miR-34a-5p-CORO1A, hsa-miR-34a-5p-FERMT3, hsa-miR-34a-5p-RAC2, hsa-miR-34a-5p-LAPTM5, hsa-miR-34c-5p-ARHGDIB, hsa-miR-34c-5p-CD48, hsa-miR-16-5p-ARHGDIB, hsa-miR-16-5p-RAC2, hsa-miR-16-5p-LAPTM5.

### Drug-hub gene interaction network analysis

Bennidazole or nifurtimox in the treatment of trypanosomiasis is the standard treatment for Chagas’ disease and has been shown to be effective in the acute phase of human trials. However, a large, prospective, multicentre, randomized study of 2854 patients with CCC in which bennidazole was used to interrupt the trypanosomiasis evaluation (BENEFIT) test was negative, showing that bennidazole did not significantly reduce chronic cardiac clinical deterioration [35]. Therefore, it is very meaningful to find new drugs that can change the prognosis of CCC.

**Fig. 7:**
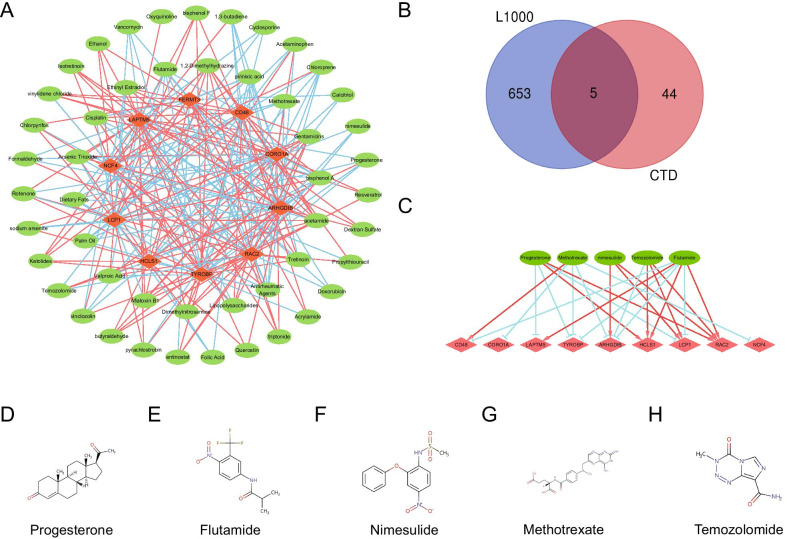
Drug-Hub gene network. **A** Drug-Hub gene Network. **B** Venn diagram of drug prediction results. **C** Co-predicted Drug-Hub gene regulatory relationships. **D**–**H** Drug chemical structure. The orange-red diamond nodes represent Hub Genes; The green elliptic nodes represent drugs; The orange-red line shows that the drug up-regulates the expression of the target gene; The blue line shows that the drug down-regulates the expression of the target gene

The hub genes were inserted into CTD and the targeted drugs that could reduce or increase the expression levels of these genes were predicted. Finally, a total of 272 target drugs were predicted. We screened drugs that simultaneously targeted three or more Hub genes and constructed a drug-hub gene interaction network with 59 nodes (49 drugs and 10 hub genes) and 249 edges (Fig. 7A). For example, LCP1, CORO1A, RAC2, ARHGDIB, FERMT3 and NCF4 are down-regulated but CD48, HCLS1, LAPTM5 and TYROBP are up-regulated by Antirheumatic Agents. Gentamicins can target 8 hub genes, CD48, HCLS1, LCP1, RAC2 are up regulated and ARHGDIB, CORO1A, LAPTM5, TYROBP are down-regulated. Quercetin can target ARHGDIB, HCLS1, LAPTM5 and down-regulated them. In addition, Acetamide, Isotretinoin, Nimesulide, Oxyquinoline and Resveratrol were identified to target these hub genes.

These hub genes were also queried using the Connectivity Map online tool. Finally, 658 targeted drugs were predicted, and five drugs, Progesterone, Flutamide, Nimesulide, Methotrexate and Temozolomide co-appeared in two drug prediction databases (Fig. 7B, C). The molecular structure of these drugs were found in the Drugbank database (Fig. 7D–H). The detail drug prediction results are displayed in Additional file 6.

## Discussion

CCC is an inflammatory dilated cardiomyopathy caused by a protozoan parasite, *Trypanosoma cruzi* [36]. In the past hundred years, a large number of studies have been conducted on CCC. However, the research on the molecular mechanism of CCC and the effective treatment methods are very limited. We use bioinformatics method to conduct data mining on CCC-related datasets, extract relevant biological information from high-dimensional data, and find the key pathways and hub genes that affect CCC occurrence. This method is the new trend of precision medical era disease research. These identified molecules have the potential to be used as disease biomarkers for patient-specific treatment and to improve the diagnosis, treatment, and prevention of CCC.

In the present study, we screened 86 Homologous genes including 73 upregulated and 13 downregulated genes which were significantly differentially expressed in the two datasets across human and mouse. GO and KEGG pathway enrichment analysis showed that these Homologous DEGs were significantly enriched in immune-related functions and pathways. These genes function in immune-related biological processes such as innate immune response, leukocyte migration, signal transduction, adaptive immune response, phagocytic vesicle membrane, Toll−like receptor binding and protein binding. Furthermore, these Homologous DEGs are involved in Natural killer cell mediated cytotoxicity, Leukocyte transendothelial migration, Chemokine signaling pathway, PI3K−Akt signaling pathway and Chagas disease (American trypanosomiasis). The PPI network of these Homologous DEGs was analyzed by String and Visualized by Cytoscape software. Based on analyzing of this network, 10 hub genes, LAPTM5, LCP1, HCLS1, CORO1A, CD48, TYROBP, RAC2, ARHGDIB, FERMT3 and NCF4 were identified. These genes are enriched in the pathway of Natural killer cell mediated cytotoxicity.

Several studies had been published about Natural killer cell mediated cytotoxicity pathway in CCC. Killer cell-mediated parasite death, which known as "microbiological programmed cell death" is similar to mammalian apoptosis and results in membrane potential decomposition, vesiculation, phospholipserine exposure, mitochondrial swelling, chromatin condensation, and DNA damage. Antimicrobial peptide granulysin (GNLY), pore-forming perforin (PFN) and granzyme (GZM) can eliminate intracellular protozoan parasites by Natural killer cell mediated cytotoxicity pathway [37]. It has been shown that infection with a virulent *T. cruzi* strain alters NK cell-mediated regulation of adaptive immune responses induced by DC cells [38]. Therefore, by intervening in this pathway, it is possible to eliminate pathogens infected cells and delay the progression of CCC. What’s more, Leukocyte transendothelial migration [39], Chemokine signaling pathway [40] and PI3K−Akt signaling pathway [41] may also be key pathways to the regulation of CCC.

As for hub genes, many studies have linked them to cardiomyopathy. For example, a genome-wide association study identifies the functional relationships between the associated variant and FERMT3 (Fermitin family homolog 3) in CCC [42]. TYROBP (TYRO protein tyrosine kinase-binding protein) shows significant increasing expression levels in model HCM (Hypertrophic cardiomyopathy) rats and affected the immune system significantly [43]. NCF4 (Neutrophil cytosol factor 4) is the component of the NADPH-oxidase. RAC2 (Ras-related C3 botulinum toxin substrate 2) can augment the production of reactive oxygen species (ROS) by NADPH oxidase. L7DG (luteolin-7-diglucuronide) pretreatment can block ISO-stimulated expression of the NCF4 and RAC2, protecting the heart against developing ISO-induced injury and fibrosis [44]. CD48 is the ligand of CD2, may facilitate interaction between activated lymphocytes and involve in regulating T-cell activation [45]. LCP1 (L-plastin, also known as plastin-2) binds to αMβ2 integrin and maintains its inactivity, thus regulating leukocyte adhesion to integrin ligand in the flow state [46]. These studies suggest that these hub genes are involved in the process of inflammatory response and myocardial remodeling and may play an important role in the development of CCC.

miRNAs are endogenous approximately 23nt RNAs that pairing with mRNAs of protein-coding genes to guide their post-transcriptional suppression [47]. These hub genes have reported to be a direct target of several miRNAs including miR-125a-5p [48], miR-375 [49], miR-125 [50], miR-183 [51]. Based on these hub genes, miRNAs (such as hsa-miR-34a-5p, hsa-miR-30d-5p, hsa-miR-27a-3p, hsa-miR-24-3p, hsa-miR-203a-3p, hsa-miR-16-5p, hsa-miR-155-5p, hsa-miR-1-3p, hsa-miR-129-2-3p and hsa-miR-124-3p) that may regulate CCC were predicted by miRNet tool. Three miRNA-related online databases, Targetscan, miRDB and miRWalk, were used to further analyze these miRNA–mRNA regulatory relationships, and three miRNAs (has-miR-34a-5p, has-miR-34c-5p, has-miR-16-5p) corresponding to hub genes were found. Many studies have linked these miRNAs to cardiomyopathy. Inhibition of miR-34a in mice attenuates moderate cardiac dysfunction by inhibiting atrial expandatrial enlargement [52]. miR-30 participates in ventricular remodeling through autophagy, apoptosis, oxidative stress and inflammation [53]. miR-24-3p regulates KEAR1-NRF2 pathway and protects myocardial cells after ischemia/reperfusion injury in mice [54]. These results demonstrated the potential roles of these miRNAs in regulating inflammation and fibrosis of the heart in CCC.

At present, Bennidazole or nifurtimox is the main treatment for Chagas’ disease. However, there is currently no effective treatment for CCC. A total of 49 Drugs (such as Gentamicins, acetamide, Isotretinoin, nimesulide, Oxyquinoline, Quercetin and Resveratrol) that may regulate CCC were predicted by CTD database. These drugs can protect the heart by regulating inflammation, autophagy, apoptosis and other pathways [55–57]. L1000 platform was used for further screening and 5 drugs (Progesterone, Flutamide, Nimesulide, Methotrexate and Temozolomide) were screened out. These drugs can serve as a starting point for follow-up studies.

The analysis of high differentially expressed homologous genes in human and mouse is helpful to understand the common characteristics and pathogenesis of CCC among species. Further experiments to verify the functions of these genes will give a more realistic picture of what happens in the human body. However, this study is based on published datasets and has some limitations. Further in vivo and in vitro studies, as well as large sample multicenter clinical studies, are required to explore better diagnostic and therapeutic methods for CCC.

## Conclusions

In conclusion, we identified there were 86 homologous DEGs in CCC between human and mouse. Bioinformatics analyses showed 122 miRNAs and 10 hub genes were associated with CCC pathogenesis via “Natural killer cell mediated cytotoxicity, Chemokine signaling pathway, Chagas disease and PI3K−Akt signaling pathway” pathways. A total of 49 drugs were predicted and 5 of them (Progesterone, Flutamide, Nimesulide, Methotrexate and Temozolomide) might be candidates for the treatment of CCC. However, the present study is a preliminary analysis, and further studies, both in vivo and in vitro, are needed to confirm these insights.

## Supplementary Information

**Additional file 1.** DEGs in GSE84796 (human) and GSE24088 (mouse)

**Additional file 2.** Homologous genes between human and mouse

**Additional file 3.** Homologous DEGs

**Additional file 4.** Function Enrichment Analysis of Homologous DEGs

**Additional file 5.** miRNAs in the miRNA-mRNA Network

**Additional file 6.** Drugs that could reduce or increase the expression levels of Hub Genes

## Data Availability

The following information was supplied regarding data availability: Data is available at NCBI GEO: GSE84796 and GSE24088.

## Abbreviations

CCC: Chronic chagasic cardiomyopathy
GEO database: Gene expression omnibus database.
DEG: Differently expressed genes
GO: Gene ontology
BP: Biological process
CC: Cellular component
MF: Molecular function
KEGG: Kyoto Encyclopedia of Genes and Genomes
DAVID: Database for annotation, visualization, and integrated discovery
PPI network: Protein–protein interaction network
MCC: Maximal clique centrality
CTD: Comparative toxicogenomics database
NC: Natural killer
DC: Dendritic cells
GNLY: Granulysin
PFN: Pore-forming perforin
GZM: Granzyme
HCM: Hypertrophic cardiomyopathy
